# Medium-vessel vasculitis following COVID-19 moderna (mRNA-1273) vaccination and the utility of PET-CT as a diagnostic tool: a case report

**DOI:** 10.1186/s41824-025-00247-7

**Published:** 2025-04-08

**Authors:** Martin H. Cherk, Luigi Zolio, Sadid Khan, Sharmayne Brady

**Affiliations:** 1 Department of Nuclear Medicine & PET Alfred Hospital, Melbourne, Australia; 2 Department of Rheumatology Alfred Hospital, Melbourne, Australia; 3 Department of Infectious Diseases Alfred Hospital, Melbourne, Australia; 4 Monash University, Melbourne, Australia; 5 University of Melbourne, Melbourne, Australia

**Keywords:** COVID-19, Vaccination, Vasculitis, Autoimmunity, Positron emission tomography, PET/CT, Imaging, 18F-FDG

## Abstract

There have been several case reports of COVID-19 “BNT162b2” (Pfizer-BioNTech) and “mRNA-1273” (Moderna) vaccination associated small and medium vessel vasculitis described in the literature however none have had ^18^F-FDG Positron Emission Tomography scans (PET/CT) performed for diagnosis. We report the case of a 57-year-old Caucasian male patient from Australia where ^18^F-FDG PET/CT scanning facilitated early detection of a medium-vessel vasculitis following Moderna (mRNA-1273) COVID-19 vaccination. The diagnosis would otherwise have been difficult and allowed exclusion of alternative diagnoses and sparing of more invasive investigations such as muscle biopsy. Our case highlights the development of a medium vessel vasculitis following mRNA based COVID-19 vaccination and demonstrates the utility of ^18^F-FDG PET/CT as an excellent non-invasive test for the detection of this serious rare and often difficult to diagnose condition.

## Introduction

mRNA based COVID-19 vaccinations such as Moderna (mRNA-1273) and Pfizer-BioNTech have been routinely recommended as a key public health measure since 2021 to assist in reducing the morbidity and mortality associated with COVID-19 infections(Mesle et al. 2024). Although uncommon, a variety of de-novo autoimmune phenomena have been reported following COVID-19 infection or vaccination(Peng et al. 2023). These autoimmune phenomena can be difficult to differentiate from other infective/inflammatory conditions and clinicians should be aware of this rare potential complication and investigations available to confirm the diagnosis.

## Case presentation

A 57-year-old Caucasian male presented to an Australian tertiary hospital with a 2-week history of progressively worsening myalgia, particularly anterior thighs, and drenching night sweats, beginning 3 days following Moderna (mRNA-1273) COVID-19 vaccination (February 2023). He had two prior Astra-Zeneca ChAdOx1-S (May & August 2021) and two prior Pfizer-BioNTech COVID-19 vaccinations (January & July 2022). There were no focal infective symptoms. There were no cranial symptoms suggestive of giant cell arteritis, cardiorespiratory, otorhinolaryngeal, gastrointestinal, neurological or cutaneous symptoms suggestive of ANCA-associated vasculitis.

Vital signs, including temperature and blood pressure, were within normal limits. Examination demonstrated mild proximal lower limb weakness (hip flexors 4/5 bilaterally) limited by pain; no rash indicative of dermatomyositis or vasculitis; cardiorespiratory, gastrointestinal and neurological examinations were unremarkable; there was no clinical cervical, axillary or inguinal lymphadenopathy or testicular pain. Targeted vascular examination showed palpable pulses in all extremities, no temporal artery tenderness or carotid, subclavian or femoral bruits. Past medical history consisted of alcohol related pancreatitis five years prior; there was no recent overseas travel.

**Fig. 1:**
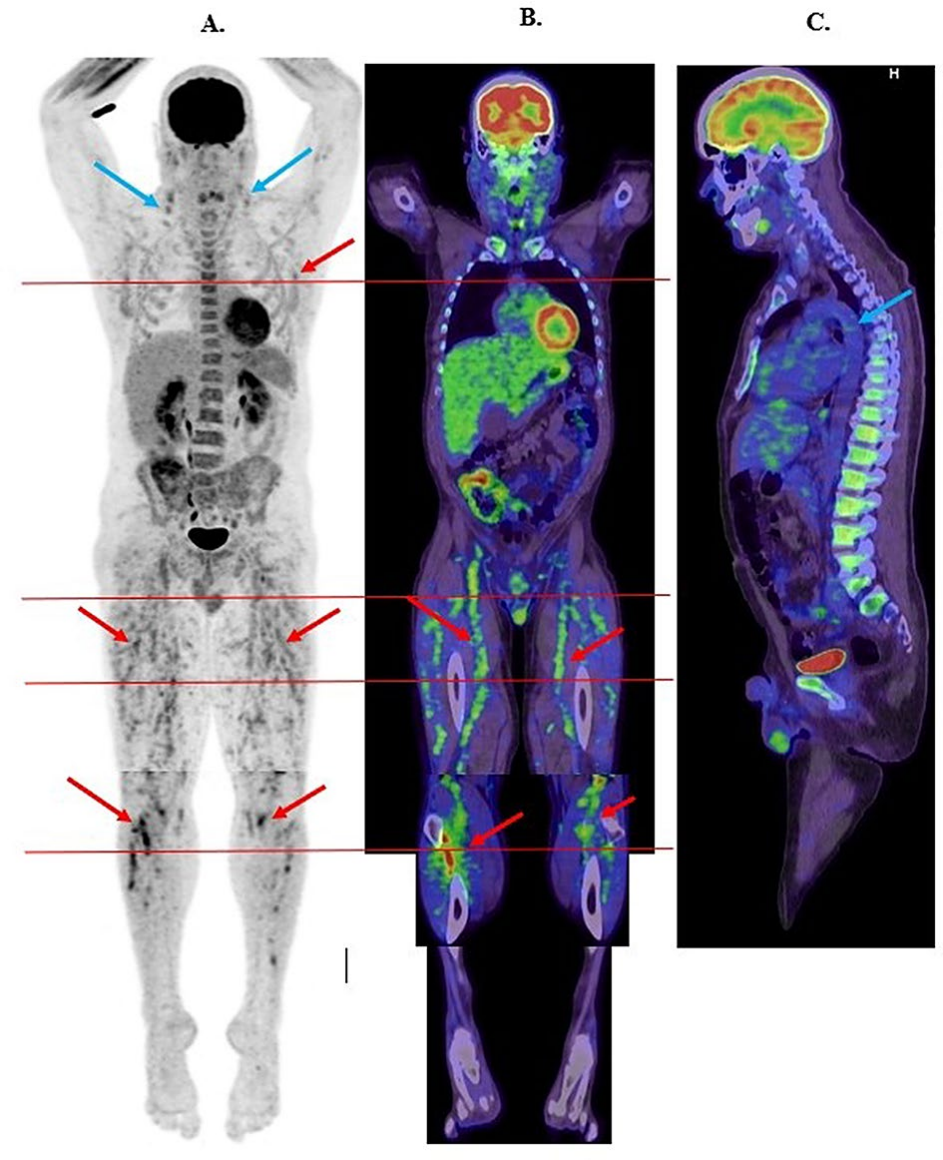
**A** Whole body ^18^F-FDG PET Maximal Intensity Projection image demonstrating widespread medium vessel vasculitis predominantly involving both lower limbs (thighs and proximal calves) and much lesser proximal upper limbs / axillae (red arrows). Moderate FDG uptake in bilateral cervical nodes also demonstrated (blue arrows). Horizontal red lines depict different trans-axial slice levels demonstrated in Fig. 2. **B** Whole body ^18^F-FDG PET/CT coronal fused images demonstrating medium vessel vasculitis involving bilateral femoral and popliteal vessels and numerous smaller branches throughout the thighs and proximal calves (red arrows). Horizontal red lines depict different trans-axial slice levels demonstrated in Fig. 2. **C** Whole body ^18^F-FDG PET/CT sagittal fused images demonstrating no abnormal FDG uptake in the ascending or descending thoracic aorta (blue arrow)

**Fig. 2:**
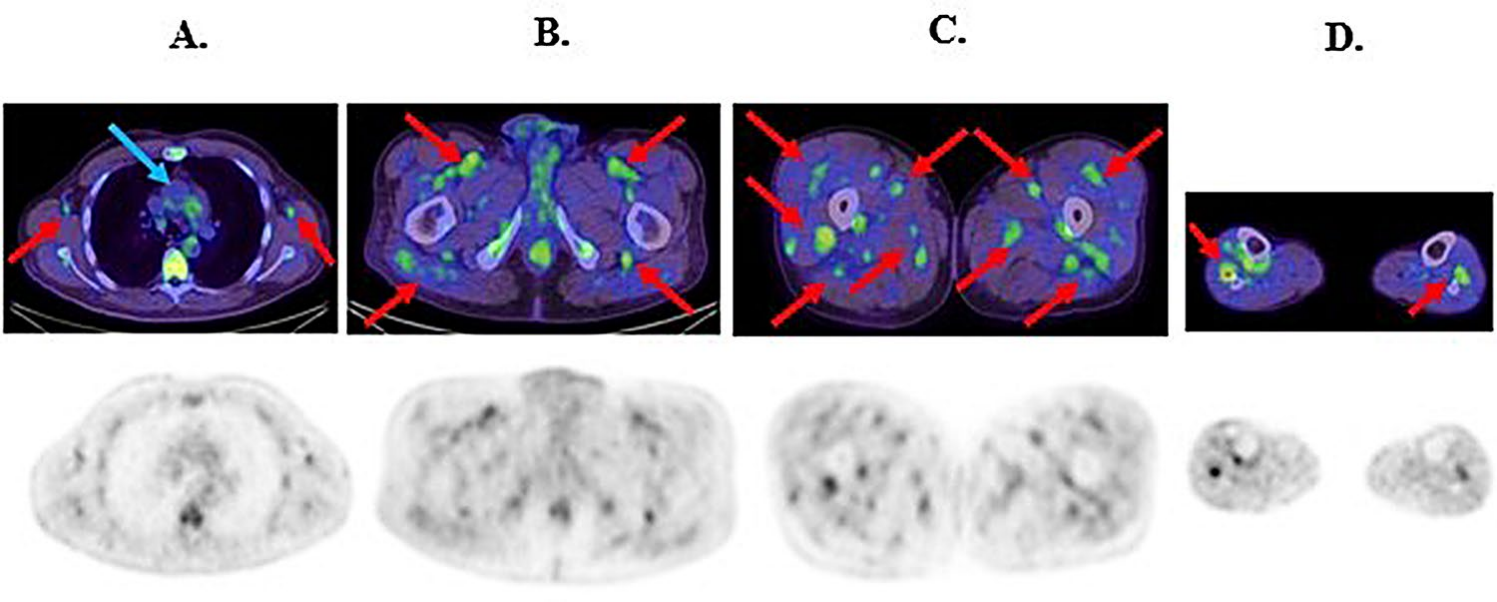
**A **^18^F-FDG PET/CT trans-axial fused and ^18^F-FDG PET images at the level of the axilla demonstrating vasculitis of bilateral axillary vessels (red arrows). No abnormal ^18^F-FDG uptake is demonstrated in the ascending aorta (blue arrow). **B **^18^F-FDG PET/CT trans-axial fused and ^18^F-FDG PET images at the level of the proximal thighs demonstrating vasculitis of bilateral femoral vessels and multiple medium sized gluteal vessels (red arrows). **C **^18^F-FDG PET/CT trans-axial fused and ^18^F-FDG PET images at the level of the mid thighs demonstrating vasculitis of bilateral popliteal vessels and multiple other medium sized vessels (red arrows). **D **^18^F-FDG PET/CT trans-axial fused and ^18^F-FDG PET images at the level of the proximal calves demonstrating vasculitis in multiple medium sized vessels (red arrows)

Blood tests demonstrated elevated inflammatory markers and liver transaminases, hypoalbuminemia and normal renal function; microbiological results and autoimmune serology were negative; urinalysis showed no significant proteinuria or haematuria (Table 1). Computed Tomography (CT) Neck and Chest was normal.^18^F-FDG Positron emission tomography scanning (PET/CT) (322 MBq ^18^F-FDG, GE Healthcare, Milwaukee USA Discovery 710 PET/CT scanner) demonstrated extensive medium vessel vasculitis throughout both thighs and to a lesser extent calves and proximal upper limbs (Fig. 1A and B). Non-enlarged moderately ^18^F-FDG bilateral cervical nodes were also demonstrated and deemed non-specific (Fig. 1A). No inflammation was demonstrated in large vessels including the thoracic aorta (Figs. 1C and 2A). No increased ^18^F-FDG uptake was demonstrated in the liver (SUVmax 2.5).

**Table 1:** Investigation results

Parameter	Result (Abnormal in Bold)	Normal Range
White Cell Count	**20.5**	3.9–12.7 × 10^9^/L
Neutrophils	**16.1**	1.9-8.0 × 10^9^/L
Platelets	**539**	150–396 × 10^9^/L
ALT	**221**	< 40 units/L
Haemoglobin	**117**	128–175 g/L
Mean Corpuscular Volume	87	80–97 fL
Blood Film	Normal	
GGT	**261**	< 55 units/L
ALP	**348**	< 110 units/L
Albumin	**23**	33–46 g/L
Urea	4.7	3.0-9.2 mmol/L
Creatinine	69	60–110 µmol/L
CRP	**350**	< 5
ESR	**75**	< 10
Blood Cultures	Negative	
EBV Serology	Negative	
CMV Serology	Negative	
HIV Serology	Negative	
Syphilis Serology	Negative	
Hepatitis B & C Serology	Negative	
Complement Levels (C3,4)	Normal	
Creatinine Kinase	75	60–285 units/L
Anti-neutrophil cytoplasmic Ab	Negative	
Rheumatoid Factor	Negative	
Cyclic citrullinated peptide Ab	Negative	
Double-stranded DNA Ab	Negative	
Liver Kidney microsome Ab	Negative	
Mitochondrial Ab	Negative	
Smooth muscle Ab	Negative	
Urinary Protein	**0.29**	0.01–0.14
Urinary Red Cells	Negative	

The patient was commenced on Prednisolone 60 mg daily with rapid clinical and biochemical improvement and was discharged 3 days later with ongoing follow-up. Myalgias, sweats, serum inflammatory markers, liver transaminases and hypoalbuminemia normalised within 21 days.

Prednisolone was weaned over a 6-month period to cessation with no recurrence of symptoms or rise in inflammatory markers during this period or during follow-up 10 months post-treatment cessation.

## Discussion

There have been several case reports of COVID-19 “BNT162b2” (Pfizer-BioNTech) and “mRNA-1273” (Moderna) vaccination associated small and medium vasculitis described in the literature(Ohmura et al. 2022; Shakoor et al. 2021; Anderegg et al. 2021) however none have had ^18^F-FDG PET/CT scans performed for diagnosis.

There is increasing evidence in the literature supporting the role of ^18^F-FDG PET/CT for non-invasive diagnosis of medium and large vessel vasculitis and the incorporation of ^18^F-FDG PET/CT as a routine investigation as part of the work up of patients suspected of having this condition(Slart et al. 2018; Ahlman and Grayson 2023).

As demonstrated in this case, ^18^F-FDG PET/CT facilitated early detection of a medium-vessel vasculitis, which otherwise would have been difficult given the negative serology, and allowed exclusion of alternative diagnoses, sparing more invasive investigations such as muscle biopsy. The utility of ^18^F-FDG-PET/CT in the evaluation of syndromes with inflammation of unknown origin is increasingly being recognised, and included in guideline recommendations for the investigation of pyrexia of unknown origin(Haidar and Singh 2022).

Our patient was diagnosed and managed as a monophasic immune-mediated vasculitis likely secondary to vaccination and he responded well, similar to one prior case report^1^. Use of other immunosuppressants or biologics was not initiated in our patient due to negative serology and relatively rapid normalization of non-specific liver function abnormalities and excellent overall clinical response to prednisolone. Notably, in other reported cases of seronegative vasculitis, the presence of renal or other end-organ involvement necessitated use of other immunosuppressants^4,8^. Cases of ANCA-associated vasculitis have been reported following COVID-19 vaccination^2^, for which most patients were treated with rituximab and/or cyclophosphamide alongside glucocorticoids at induction in keeping with established treatment guidelines (Hellmich et al. 2007), irrespective of a potential causative relationship of prior vaccination.

Hepatitis B Serology was negative and there were no clinical features such as skin lesions, livedo or testicular pain and no saccular aneurysms were demonstrated on diagnostic CT to suggest Polyarteritis Nodosa. The lack of significant bone marrow dysfunction including thrombocytopenia and macrocytic anaemia, one of the hallmarks of VEXAS syndrome along with the monophasic nature of the illness suggested VEXAS syndrome was unlikely.

We acknowledge a limitation of this case report is that no biopsies were performed to enable histopathological confirmation of vasculitis however given the striking ^18^F-FDG PET/CT scan findings typical for a widespread medium vessel vasculitis and rapid response to treatment, the patient was not subject to biopsy and its inherent risks.

Multiple types of de-novo autoimmune phenomena have been reported following COVID-19 infection or vaccination (Peng et al. 2023). A specific mechanism of mRNA-based COVID-19 vaccine-induced vasculitis is uncertain, however potential mechanisms for vaccine-induced autoimmunity have been postulated. A genetic predisposition has been proposed, given autoimmunity only occurs in a minority of individuals (Chen et al. 2022). Molecular mimicry of vaccine products with self-antigens may lead to autoreactive T cells, polyclonal B-cell activation, and autoantibody formation (Hromić-Jahjefendić et al. 2023). Potential culprits for molecular mimicry include the Spike (S) protein (the main antigen of most COVID-19 vaccines) or adjuvants (Chen et al. 2022). Vaccine adjuvants or mRNA are identified by endosomal toll-like receptors, which may trigger the NLR pyrin domain containing 3 (NLRP3) inflammasome, leading to multiple downstream cytokine activation (Teijaro and Farber 2021).

This report of a possible rare vaccination side effect does not diminish the established health benefits at a population level of COVID-19 vaccination, including mortality reduction (Mesle et al. 2024). Importantly, a recent cohort study found that prior vaccination may attenuate the risk of COVID-19 infection-associated autoimmunity (Peng et al. 2023).

In summary, our case highlights the development of a monophasic medium vessel vasculitis following COVID-19 vaccination and demonstrates the excellent utility of ^18^F-FDG PET/CT for the detection of medium vessel vasculitis and exclusion of alternative diagnoses in this setting. ^18^F-FDG-PET/CT should be considered early as a potentially useful non-invasive investigation in patients suspected to have this condition following mRNA based COVID-19 vaccinations or COVID-19 infection.

## Data Availability

Not applicable.

## Declarations

### Author information

MC – Nuclear Medicine Physician & Medical Oncologist (FRACP, Australia) LZ – Rheumatologist (FRACP, Australia), SK – Infectious Diseases Physician (FRACP, Australia), SB – Rheumatologist (FRACP, Australia).
